# Evidence of aquaporin involvement in human central pontine myelinolysis

**DOI:** 10.1186/2051-5960-1-40

**Published:** 2013-07-25

**Authors:** Bogdan F Gh Popescu, Reem F Bunyan, Yong Guo, Joseph E Parisi, Vanda A Lennon, Claudia F Lucchinetti

**Affiliations:** 1 Department Anatomy and Cell Biology, University of Saskatchewan, Saskatoon, SK, Canada; 2 Cameco MS Neuroscience Research Center, University of Saskatchewan, Saskatoon, SK, Canada; 3 Department of Neurology, Neurosciences Center, King Fahad Specialist Hospital, Dammam, Saudi Arabia; 4 Department of Neurology, Mayo Clinic, College of Medicine, 200 First St. SW, Rochester 55905, MN, USA; 5 Department of Laboratory Medicine and Pathology, Mayo Clinic, Rochester, MN, USA; 6 Department of Immunology, Mayo Clinic, Rochester, MN, USA

**Keywords:** Osmotic demyelination syndrome, Astrocyte, Demyelination

## Abstract

**Background:**

Central pontine myelinolysis (CPM) is a demyelinating disorder of the central basis pontis that is often associated with osmotic stress. The aquaporin water channels (AQPs) have been pathogenically implicated because serum osmolarity changes redistribute water and osmolytes among various central nervous system compartments.

**Results:**

We characterized the immunoreactivity of aquaporin-1 and aquaporin-4 (AQP1 and AQP4) and associated neuropathology in microscopic transverse sections from archival autopsied pontine tissue from 6 patients with pathologically confirmed CPM. Loss of both AQP1 and AQP4 was evident within demyelinating lesions in four of the six cases, despite the presence of glial fibrillary acidic protein (GFAP)-positive astrocytes. Lesional astrocytes were small, and exhibited fewer and shorter processes than perilesional astrocytes. In two of the six cases, astrocytes within demyelinating lesions exhibited increased AQP1 and AQP4 immunoreactivities, and gemistocytes and mitotic astrocytes were numerous. Blinded review of medical records revealed that all four cases lacking lesional AQP1 and AQP4 immunoreactivities were male, whereas the two cases with enhanced lesional AQP1 and AQP4 immunoreactivities were female.

**Conclusions:**

This report is the first to establish astrocytic AQP loss in a subset of human CPM cases and suggests AQP1 and AQP4 may be involved in the pathogenesis of CPM. Further studies are required to determine whether the loss of AQP1 and AQP4 is restricted to male CPM patients, or rather may be a feature associated with specific underlying precipitants of CPM that may be more common among men. Non-rodent experimental models are needed to better clarify the complex and dynamic mechanisms involved in the regulation of AQPs in CPM, in order to determine whether it occurs secondary to the destructive disease process, or represents a compensatory mechanism protecting the astrocyte against apoptosis.

## Background

Central pontine myelinolysis (CPM) is a demyelinating disorder affecting the central basis pontis [1,2]. Lesions characteristically exhibit apoptotic cells, loss of oligodendrocytes, microgliosis, astrogliosis, preservation of neurons and axons, and infiltration by macrophages [3-10]. Inflammation usually is lacking, but the presence of mild lymphocytic inflammatory infiltrates has been reported and does not exclude the diagnosis [11].

Rapid correction of chronic hyponatremia is a known cause of CPM, but the molecular pathogenesis remains elusive [3,4,6,7,12]. CPM occurs in the context of severe illness, and is commonly a complication of conditions with altered serum Na^+^ levels: alcoholism, liver transplantation, malnutrition, hepatic cirrhosis, burns or the syndrome of inappropriate antidiuresis (SIAD) [11,13-16]. Recognition that CPM also occurs when other serum osmolytes are altered has prompted the use of “osmotic demyelination syndrome” as alternative terminology [17].

Changes in serum osmolarity are recognized to cause cerebral edema and redistribution of water and inorganic and organic osmolytes among various central nervous system (CNS) compartments [18-21]. Astrocytes, which are five times more abundant than neurons in the CNS, enwrap synapses and blood vessels and participate in blood–brain barrier maintenance. They are therefore in a unique and critical position for controlling brain volume changes [22,23]. Aquaporins (AQPs) are key regulators of brain volume homeostasis. While aquaporin-4 (AQP4) has been considered the major CNS water channel and is confined to astrocytes and ependyma [24,25], recent studies have shown that astrocytes also express aquaporin-1 (AQP1) in human and non-human primate brains [26-28]. However the complementary and/or redundant roles of astrocytic AQP1 and AQP4 in regulation of water homeostasis in the human CNS have yet to be addressed. This study is the first to systematically characterize astrocyte pathology and expression of AQP1 and AQP4 proteins in human CPM lesions.

## Results

### General neuropathological characteristics of CPM lesions

**Figure 1:**
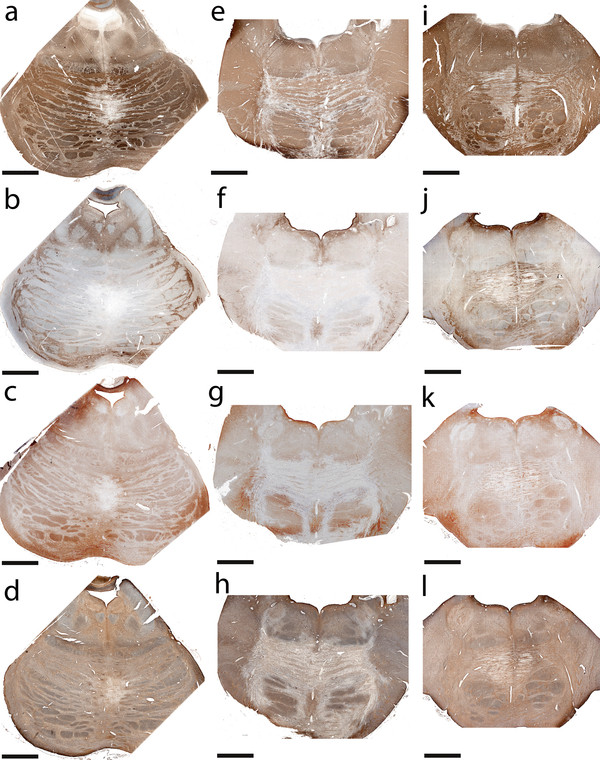
**AQP1, AQP4 and GFAP protein expression in human CPM lesions. (a-d)** A demyelinated lesion in the central *basis pontis***(a)** of a 56-year-old man with CPM in context of rapidly corrected hyponatremia shows loss of astrocytic AQP4 extending beyond the area of demyelination **(b)**, loss of AQP1 **(c)**, but retained GFAP **(d)**; **(e-h)** A CPM lesion in the central *basis pontis* of a 53-year-old man with CPM and severe dehydration, malnutrition and hypernatremia shows relative preservation of myelin **(e)**, loss of astrocytic AQP4 **(f)**, AQP1 **(g)**, but preserved GFAP **(h)**; **(i-l)** A demyelinated lesion in the central *basis pontis***(i)** of a 24-year-old woman with CPM in the context of hepatic failure and hypokalemia shows increased AQP4 **(j)**, AQP1 **(k)** and GFAP expression **(l)**; **(a, e, i)** PLP; **(b,f, j)** AQP4; **(c, g, k)** AQP1; **(d, h, l)** GFAP; (scale bar = 5 mm).

**Figure 2:**
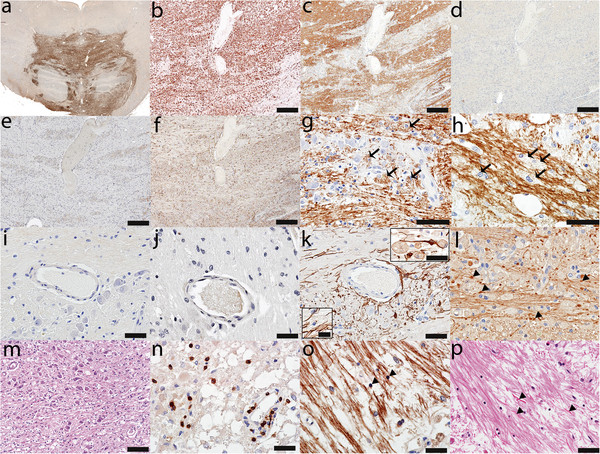
**CPM lesions with loss of AQPs. (a)** The demyelinated lesion in the central *basis pontis***(e-h)** is heavily infiltrated with activated macrophages (KiM1P, scale bar = 5 mm); **(b)** Higher magnification of an area of macrophage infiltration (KiM1P, scale bar = 500 μm) also shows **(c)** relative myelin preservation (PLP, scale bar = 500 μm), **(d)** loss of AQP4 (AQP4, scale bar = 500 μm), **(e)** loss of AQP1 (AQP1, scale bar = 500 μm), but **(f)** preserved GFAP immunoreactivity (GFAP, scale bar = 500 μm); Despite preservation of myelin, the presence of macrophages containing myelin degradation products both in the **(g)** gray matter (PLP, scale bar = 50 μm) and **(h)** white matter (PLP, scale bar = 50 μm) is consistent with active demyelination; The normal perivascular distribution of **(i)** AQP4 (AQP4, scale bar = 50 μm) and **(j)** AQP1 (AQP1, scale bar = 50 μm) is lost, but **(k)** small GFAP-immunoreactive astrocytes with short processes are still present in lesions; upper inset shows such an astrocyte in close contact with two macrophages; lower inset shows a macrophage containing GFAP + degradation products (GFAP, scale bar = 50 μm, upper inset scale bar = 25 μm, lower inset scale bar = 12.5 μm); **(l)** Axons are preserved, but the presence of axonal swellings (arrows) indicates axonal injury (NF, scale bar = 50 μm); **(m)** Neurons are relatively well preserved (HE, scale bar = 50 μm); **(n)** The lesion also contains mild parenchymal and perivascular T-cell inflammation (CD3, scale bar = 50 μm); **(o)** Apoptotic oligodendrocytes (arrowheads) are present (PLP, scale bar = 50 μm); **(p)** Rosenthal fibers are also present (HE, scale bar = 100 μm).

**Figure 3:**
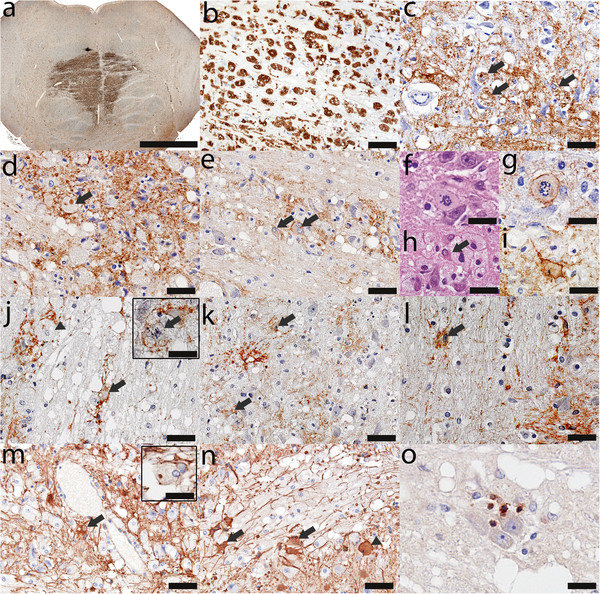
**CPM lesions with increased expression of AQPs. (a)** The demyelinated lesion in the central *basis pontis* (Figure 2i-l) is heavily infiltrated with activated macrophages (KiM1P, scale bar = 5 mm); **(b)** Higher magnification of an area of macrophage infiltration (KiM1P, scale bar = 100 μm) also shows **(c)** relative myelin preservation and myelin containing macrophages (arrows) consistent with active demyelination (PLP, scale bar = 50 μm); **(d, e)** The lesion shows increased AQP4 expression, occasional macrophages containing AQP4-immunoreactive degradation products (**d**, arrow), AQP4-positive gemistocytic astrocytes (**e**, arrows) (AQP4, scale bar = 50 μm); **(f)** Mitotic astrocytes are present (HE, scale bar = 25 μm) and **(g)** AQP4 immunoreactive (AQP4, scale bar = 25 μm); **(h)** Gemistocytes are also present (HE, scale bar = 25 μm), and **(i)** AQP4 immunoreactive (AQP4, scale bar = 25 μm); **(j, k, l)** The lesion shows increased AQP1 expression, occasional macrophages containing AQP1-immunoreactive degradation products (**j**, arrow head), AQP1-positive gemistocytic astrocytes (**j**, **k**, **l**, arrows) and AQP1-positive mitotic astrocytes (**j**, inset arrow) (AQP1, scale bar = 50 μm); **(m, n)** The lesion shows increased GFAP immunoreactivity, tissue vacuolation, occasional macrophages containing GFAP + degradation products (**m**, inset), GFAP + gemistocytic astrocytes (**m**, **n**, arrows) as well as GFAP + mitotic astrocytes (**n**, arrow head) (GFAP, scale bar = 50 μm, inset scale bar = 25 μm); **(o)** Parenchymal inflammation is also present (CD3, scale bar = 25 μm).

All lesions identified in CNS tissues examined from the 6 CPM patients exhibited active demyelination (Figure 1a, e, i; Figure 2g, h; Figure 3c). Lesions affected the base of the central pons symmetrically (Figure 1a, e, i; Figure 2a; Figure 3a). There was relative preservation of axons and neurons (Figure 2l, m) and, to some extent, of myelin (Figure 1e, i; Figure 2c, g, h; Figure 3c). All lesions were heavily infiltrated by activated macrophages (Figure 2a, b; Figure 3a, b) containing abundant myelin degradation products, a hallmark of active demyelination (Figure 2g, h; Figure 3c). There was a mild degree of T lymphocyte infiltration around vessels and in the parenchyma (Figure 2n; Figure 3o), and evidence of axonal swelling (Figure 2l) and oligodendrocyte apoptosis (Figure 2o). No deposits of complement activation products were found.

**Figure 4:**
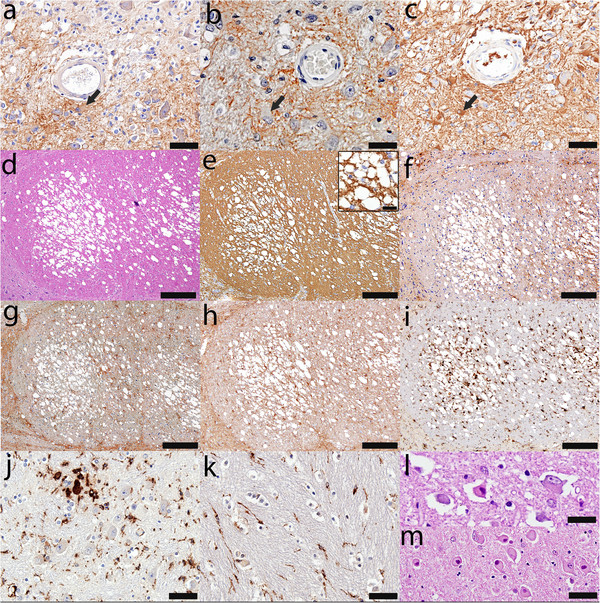
**Neuropathology of perilesional areas.** Gemistocytes (arrows in **a-c**) are present and the expression of **(a)** AQP4 (AQP4, scale bar = 50 μm), **(b)** AQP1 (AQP1, scale bar = 50 μm), and **(c)** GFAP (GFAP, scale bar = 50 μm) is increased at the lesions’ borders. **(d)** Vacuolated white matter (HE, scale bar = 250 μm) with the appearance of **(e)** intramyelinic edema (inset shows higher magnification) is a common feature of nonlesional pontine white matter tracts with preserved myelin (PLP, scale bar = 250 μm, inset scale bar = 25 μm), **(f)** normal AQP4 (AQP4, scale bar = 250 μm), **(g)** normal AQP1 (AQP1, scale bar = 250 μm), **(h)** normal GFAP expression (GFAP, scale bar = 250 μm), and **(i)** microglial infiltration (KiM1P, scale bar = 250 μm); **(j)** Normal appearing gray and **(k)** white matter show profound microglial activation with rare clusters of small macrophages (KiM1P, scale bar = 50 μm); Red ischemic neurons **(l)** and ballooned neurons **(m)** are present in the normal appearing gray matter (HE, scale bar = 50 μm).

Nonlesional pontine white matter tracts were frequently vacuolated (Figure 4d-i), compatible with intramyelinic edema (Figure 4e, inset), and were infiltrated by microglia (Figure 4i). However, myelin was preserved (Figure 4b), and AQP4 (Figure 4f), AQP1 (Figure 4g) and GFAP (Figure 4h) immunoreactivities were of normal intensity. In normal appearing gray and white matter there was evidence of prominent microglial activation (Figure 4j, k), rare clusters of small macrophages (Figure 4j), and ballooned and red ischemic neurons (Figure 4l, m).

### Expression of AQP1 and AQP4 in the normal pons

**Figure 5:**
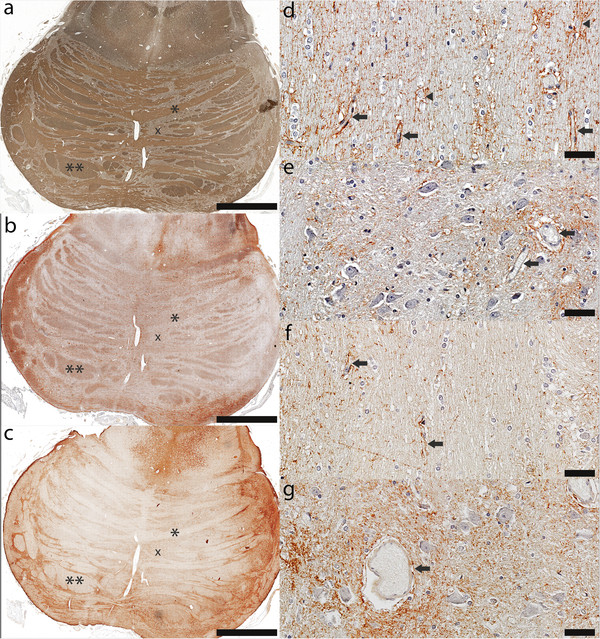
**Distribution of AQP1 and AQP4 in the control pons. (a)** Myelin immunohistochemistry reveals the characteristic features of the *basis pontis*: transverse white matter pontocerebellar tracts (*) and descending white matter corticospinal and coticobulbar tracts (**) intermingled with pontine grey matter nuclei (x) (PLP, scale bar = 5 mm); **(b)** Pontine white matter astrocytes preferentially express aquaporin 1 (AQP1) (AQP1, scale bar = 5 mm); **(c)** Astrocytes in the pontine nuclei preferentially express aquaporin 4 (AQP4) (AQP4, scale bar = 5 mm); **(d)** AQP1 in the white matter is highly concentrated on the membrane of the astrocytic cell bodies (arrow heads) and in all astrocytic foot processes, including those abutting blood vessels (arrows) (AQP1, scale bar = 100 μm); **(e)** AQP1 immunoreactivity in the pontine nuclei is preferentially observed around blood vessels (arrows) and around some, but not all neurons (AQP1, scale bar = 50 μm); **(f)** AQP4 immunoreactivity in the white matter is mainly observed around blood vessels (arrows) (AQP4, scale bar = 100 μm); **(g)** AQP4 positive astrocytic processes in the pontine nuclei envelop the blood vessels (arrow) as well as all neuronal cell bodies (AQP4, scale bar = 50 μm).

Astrocytes in the normal pons expressed both AQP1 and AQP4 (Figure 5). No differences were observed between the female and male controls.

Astrocytes in the pontine white matter tracts expressed preferentially, but not exclusively, AQP1 (Figure 5a-c). AQP1 in the white matter was highly concentrated on the membrane of the astrocytic cell bodies and in all astrocytic foot processes, including those abutting blood vessels (Figure 5d). AQP4 immunoreactivity in the white matter was mainly observed in astrocytic foot processes abutting blood vessels (Figure 5f).

Astrocytes in the pontine grey matter nuclei preferentially, but not exclusively, expressed AQP4 (Figure 5a-c). AQP4 positive astrocytic processes enveloped the blood vessels as well as the neuronal cell bodies (Figure 5g). AQP1 immunoreactivity in the pontine nuclei was preferentially observed around blood vessels and around some, but not all neurons (Figure 5e).

### Astrocytic pathology in CPM lesions

Loss of AQP1 and AQP4 was evident within demyelinating lesions in four of the six cases (Table 1, Figure 1b, f, c, g; Figure 2d, e). Areas with loss of AQPs (Figure 1b, c, f, g; Figure 2d, e) corresponded to areas of demyelination (Figure 1a, e; Figure 2d, e) and macrophage infiltration (Figure 2a, b), and AQP immunoreactivity was enhanced at the lesion borders (Figure 4a, b). All four cases demonstrated a loss of the characteristic perivascular distribution of AQP4 (Figure 2i) and AQP1 (Figure 2j) within the area of active demyelination. Beyond the demyelinating lesions, AQP1 and AQP4 immunoreactivities were preserved (Figure 1b, c, f, g). Lesions uniformly retained GFAP-immunoreactive astrocytes, despite AQP1 and AQP4 loss (Figure 2f, k), but astrocyte numbers were reduced. Lesional astrocytes were smaller and had fewer and shorter processes (Figure 2k) than perilesional astrocytes (Figure 4c), giving the impression of GFAP loss within lesions when sections were observed under low magnification (Figure 1d, h). Gemistocytes were rare within lesions, but were abundant at lesion borders (Figure 4a-c). The nuclear condensation and fragmentation typical of apoptosis was not observed in either lesional or non-lesional astrocytes. Occasional macrophages contained GFAP-positive material (Figure 2k, lower inset), and Rosenthal fibers were present (Figure 2p).

**Table 1:** Demographic and clinical characteristics of CPM patients

**No.**	**Age**	**Sex**	**AQP1/4 tissue**	**Brief history**	**Probable cause/**
	**(years)**		immunoreactivity*		**underlying cause of**
					**CPM**
1	56	M	0	Alcoholism; nausea and vomiting 1 week; hyponatremia, abnormal liver function tests; presented to Emergency Room in cardiorespiratory arrest.	Rapid correction of hyponatremia
2	53	M	0	Depression and chronic obstructive pulmonary disease. Not seen for 4 days; found dehydrated and malnourished; drug and alcohol screen negative.	Dehydration, malnutrition and hypernatremia
3	33	M	0	Cryptogenic cirrhosis with antitrypsin heterozygosity; orthotopic liver transplant and hyponatremia followed by progressive obtundation and seizures.	Orthotopic liver transplant
4	45	M	0	Small-cell lung carcinoma, metastatic to liver; abnormal liver function tests; possible malnutrition; hyperkalemia; several syncopal episodes in preceding hours; presented with respiratory failure.	SIAD secondary to small-cell lung carcinoma
5	24	F	↑	Hepatic failure and autoimmune thrombocytopenic purpura, 6 months; altered consciousness level and nonconvulsive status epilepticus 5 weeks before death; hypocalcemia, hyperammonemia and elevated liver enzymes.	Hepatic failure and hypocalcemia
6	68	F	↑	Hypertension, obesity, diabetes mellitus, hyperuricemia and remote endometrial carcinoma (treated by resection, chemotherapy, and radiotherapy); presented with gangrene of the right fifth toe; generalized seizure followed by unresponsiveness.	Diabetes mellitus type II

*0 = absent; ↑ = increased.

In two of the six cases astrocytes within demyelinating lesions exhibited enhanced AQP1 and AQP4 immunoreactivity (Table 1; Figure 1j, k; Figure 3d-g, j-l). In contrast to lesions with loss of AQPs, these lesions exhibited astroglial activation, numerous gemistocytes, and abundant mitoses (Figure 3d-l), as well as pronounced GFAP immunoreactivity (Figure 3m, n). Occasional macrophages contained in their cytoplasm degradation products of AQP4 (Figure 3d), AQP1 (Figure 3j) and GFAP (Figure 3m).

### Demographics and clinical characteristics of the CPM patients (Table 1)

Blinded review of medical records revealed that all 4 CPM cases with lesional loss of AQP1 and AQP 4 immunoreactivities were male, whereas the 2 cases with increased lesional AQP1 and AQP4 immunoreactivities were female. Median age at hospitalization was 49 years (range 24–68). Median disease duration (interval to death from arrival at hospital or from clinical onset of CPM) was 9 days (range 1–60).

All cases had clinical evidence of an osmotic disturbance or had an underlying condition which predisposed to the development of CPM (Table 1). Patient 1 had severe hyponatremia in the setting of alcoholism. In correcting the hyponatremia, serum Na^+^ increased by 2 mEq/L in the first 24 hours and by another 12 mEq/L in the next 24 hours (Figure 1a-d) [3,6,11-16]. Patient 2 had malnutrition, severe dehydration, and hypernatremia (172 mEq/L) (Figure 1e-h; Figure 2) [11,13-16]. Patient 3 had hyponatremia following liver transplantation [11,13-16,29-31]. Patient 4 had small-cell lung carcinoma with brain and liver metastases, abnormal liver function tests, hyperkalemia, hyperphosphatemia and SIAD (presumed to be paraneoplastic) as the precipitating factor for CPM [11,14]. Patient 5 had hepatic failure and hypokalemia (Figure 1i-l; Figure 3) [1,11,13-16,32]. Patient 6 had diabetes mellitus type II [11,13-16,33,34].

## Discussion

### CPM lesions are characterized by astrocytopathy

Apoptosis and loss of oligodendrocytes are traditionally considered the pathological hallmarks of CPM. However we found in a subset of CPM cases that astrocytes were variably reduced in numbers, were small with fewer and shorter processes, and AQP1 and AQP4 immunoreactivities were lost within actively demyelinating regions with relative preservation of myelin. These findings suggest that prominent degenerative changes in astrocytes may precede demyelination. Astrocyte damage has been described in a single previous report [8], however this is the first study characterizing in detail the predominant astrocytic injury in human CPM. Although autopsy studies do not permit definite determination of whether this astrocytic damage is primary or secondary, a rat model of osmotic demyelination suggests that rapid correction of hyponatremia triggers astrocytic damage in male rats, and that ensuing loss of trophic communication between astrocytes and oligodendrocytes results in inflammation, microglial activation, oligodendrocyte injury, and subsequent demyelination [35]. The constellation of neuropathological findings we observed, particularly among four of the CPM patients, implies a similar sequence of pathogenic events, with two notable differences. First, GFAP immunoreactivity is lost in rat lesional astrocytes, but is retained in lesional astrocytes of patients with CPM, despite a loss of both AQP1 and AQP4. This argues against the loss of AQPs being strictly due to a loss of astrocytes. Second, in the rat CPM model, lesional astrocytes show signs of extensive apoptosis, whereas apoptotic astrocytes were not evident in human CPM. Interspecies and interregional astrocytic heterogeneity may explain these discrepancies. Most human CPM lesions reside in the pons, while rodent CPM lesions typically reside in the external capsule, claustrum, corpus striatum, neocortex, hippocampus and anterior commissure [3,4]. Furthermore, unlike rodent astrocytes which only express AQP4, astrocytes in the human brain express both AQP1 and AQP4 [27,28]. Species and CNS differences with respect to astrocyte morphology, properties and functions therefore exist and have been described previously in both the normal and diseased brain [36-38].

### Relevance of AQPs to CPM

A unique finding in our study is that immunoreactivity for AQP proteins is lost in some actively demyelinating lesions in human CPM. This finding was not universal. Two of the six cases had an increase of AQP1 and AQP4 immunoreactivities within actively demyelinating CPM lesions. Complex dynamic processes that maintain water homeostasis likely contribute to the discordance of these findings. As the principal water channels in human CNS astrocytes, both AQP1 and AQP4 are involved indirectly in osmolyte movement through functional and molecular interactions with numerous ion channels and osmolyte transporters [18-21].

The observed loss of AQP1 and AQP4 in four of six cases may reflect a compensatory astrocytic response to a hypotonic milieu. The astrocyte response anticipated in a hypotonic environment is swelling due to osmotically-driven water influx through AQP channels. The astrocyte’s physiological regulatory volume decrease preserves cell volume homeostasis by releasing inorganic and organic osmolytes, which in turn are followed by water [19,20,39,40]. The therapeutic use of a hyperosmolar solution to rapidly correct chronic hypotonicity causes endothelial cell shrinkage and blood–brain barrier disruption, vasogenic edema and increased osmolarity of the CNS extracellular space [41,42]. Water efflux from astrocytes to compensate for intracellular and extracellular osmolarities, further accentuates the shrinkage of astrocytes. Loss of AQP4 and AQP1 could represent a protective mechanism whereby astrocytes restrict water loss and prevent the triggering of apoptosis precipitated by a loss of cell volume [40,43-49]. The reduced number and small size of GFAP-positive (non-apoptotic) astrocytes that we observed in regions of AQP1 and AQP4 loss within CPM lesions are compatible with this hypothesis.

In contrast, two of the six CPM cases were characterized by astrogliosis associated with increased AQP1 and AQP4 immunoreactivities. In the setting of vasogenic edema accompanying CPM, an increase in AQP1 and AQP4 should facilitate water removal [21,50]. Administration of urea or reinduction of hyponatremia during inadvertent rapid correction of hyponatremia are both known to decrease the number and severity of CPM lesions and to increase AQP4 expression [51-56]. However, AQPs are only one of several factors that may be involved in the genesis and resolution of CPM. Although this was a small series, we did not observe differences between the severity of lesions, nor did the clinical outcomes differ in the patients regardless of observed tissue AQP status.

### Potential pathogenic consequences of AQPs loss in CPM

AQP4 co-localizes and acts in concert with the inwardly-rectifying Kir4.1 K^+^ channel [57-65], the K^+^ clearing electro neutral co-transporter NKCC [66], volume regulated anion channels [67], Ca^2+^ activated K^+^ channels [68], the Kv1.5 voltage-gated K^+^ channel [69], two-pore domain K^+^ channel [70,71], transient receptor potential vanniloid related channel 4 [72] and excitatory amino acid transporter 2 (EAAT2) [73]. Although the co-localization of AQP1 with ion channels on CNS astrocytes has not been studied so far, AQP1 is known to co-localize with the Na-K-2Cl cotransporter in meningiomas [74], and with the α-epithelial Na(+) channel in the lung [75]. Therefore, loss of AQP1 and AQP4 in CPM lesions would be expected to not only impair bidirectional water fluxes in astrocytes, but could also impair the transport of ions and organic osmolytes, and possibly aggravate osmotic stress [76] and glutamate excitotoxicity [73].

### Differences in the expression of AQPs in CPM lesions

An unexpected finding in our study was that all CPM cases exhibiting lesional loss of AQP1 and AQP4 immunoreactivity were male patients and that female cases exhibited a lesional increase in astrocytic AQP immunoreactivity. The small number of patients precludes definitive conclusions concerning sexual dimorphism in astrocytic AQP regulation. However, female and male sex hormones have been reported to have opposite effects on ion transporters, and therefore could exert similar effects on the expression of water channels. For instance, it is known that estrogens impair brain adaptation to hyponatremia whereas androgens enhance it through respective inhibition and stimulation of Na^+^-K^+^-ATPase [77-84]. Furthermore, it is known that estrogen induces AQP1 expression by activating the estrogen-response element in the promoter of the Aqp1 gene during angiogenesis in human breast and endometrial carcinomas [85]. Thus, it is conceivable that in the setting of osmotic stress, estrogens and androgens could have opposite effects on AQP1 and AQP4 expression in female and male patients. If downregulation of AQP1 and AQP4, and/or failure of their upregulation were an androgen-dependent mechanism, this would, on the one hand protect the astrocyte from apoptosis, but also be expected to worsen the osmotic disturbance. This outcome could plausibly explain the higher incidence of CPM observed in the male population [5,16].CPM is also known to occur in association with a multitude of several underlying conditions, with alcoholism, liver transplantation, malnutrition, hepatic cirrhosis, burns and SIAD being among the most common. Although most of these conditions cause changes in serum Na^+^ levels, changes in other serum osmolytes have also been associated with CPM including hypokalemia, hyperglycemia, hypophosphatemia, and correction of hyperammonemia [11,13-16]. Furthermore, hypoxia and arginine vasopressin, in addition to estrogen, affects the brain’s ability to adapt to cellular edema [39]. Therefore the differences observed in the expression of AQP1 and AQP4 among the human CPM lesions may alternatively be explained by different underlying disease processes that contributed to the development of CPM.

### AQP4 in CPM and NMO

Our findings, corroborated by prior studies, justify considering CPM a primary astrocytopathy with secondary demyelination [86-88]. Furthermore, we demonstrate for the first time that AQP1 and AQP4 contribute to human CPM pathology and pathogenesis. A primary role for AQP4 has been associated with neuromyelitis optica (NMO), an autoimmune inflammatory astrocytopathy caused by complement-activating IgG autoantibodies directed against AQP4 [88-90]. Early active demyelinating lesions in both NMO and a subgroup of CPM patients are characterized by a spectrum of astrocytic damage, AQP4 loss, retention of small GFAP immunoreactive astrocytes with fewer and shorter processes, intramyelinic edema and apoptosis of oligodendrocytes with secondary demyelination. Loss of AQP4 in NMO is caused in part by the internalization of AQP4 by some astrocytes following its interaction with NMO-IgG (antigenic modulation). Complement, when available, is activated by NMO-IgG remaining on internalization-resistant AQP4 [88-91] expressing astrocytes. In contrast to previously published studies [92], we did not find evidence for either complement activation or AQP4 astrocyte internalization in CPM lesions, suggesting that loss of AQP4 can occur in the absence of complement-activating antibodies. The CPM lesions characterized by AQP4 and AQP1 loss, but preserved GFAP staining of astrocytes in the absence of antibodies, complement activation or AQP4 astrocyte internalization, somewhat resemble the type 6 NMO lesions recently described which show a more pronounced loss of AQP4 and AQP1 than GFAP, and also occur in the absence of immunoglobulin deposition or complement activation [27].

We propose that loss of AQP4 and AQP1 in the setting of CPM may be due to compensatory changes in response to the local osmotic environment, resulting in astrocytic injury as a consequence of the osmotic stress. Type 6 NMO lesions may similarly mirror astrocytic injury caused by osmotic stress due to disturbances of the local osmotic environment either due to a possible direct effect of NMO-IgG on water transport [90] or triggered by astrocytes in nearby regions that are damaged via antibody- and complement-mediated mechanisms [88-91].

## Conclusions

Our findings provide the first evidence that astrocytic AQP1 and AQP4 may be involved in the pathogenesis of CPM. Both are lost in a subset of CPM patients. It remains to be determined whether loss of AQPs in CPM is a protective compensatory mechanism to protect against astrocytic apoptosis. Furthermore, additional studies are needed in order to clarify whether AQP loss is a pathological characteristic of all male patients with CPM, or rather a feature associated with specific underlying causes of CPM. Since studies of autopsied human tissues offer only a single snapshot in disease evolution and since there are major differences between rodent and human astrocytes, non-rodent models are needed in order to better define the complex and dynamic relationship between regulation of AQPs and astrocyte injury in CPM.

## Methods

### Archival material

To characterize AQP1 and AQP4 immunoreactivity and neuropathological characteristics of CPM, we analyzed microscopic transverse sections of the pons in archival autopsied tissue from two control cases (17 year old female and 15 year old male), and six pathologically confirmed CPM cases. Clinical information was obtained from medical records. The study was approved by the Institutional Review Board of the Mayo Clinic, Rochester.

### Neuropathological evaluation and immunohistochemistry

Specimens were fixed in 10–15% formalin and embedded in paraffin. Sections were stained with haematoxylin and eosin (HE) for morphological evaluation and Luxol-fast blue-periodic acid-Schiff (LFB/PAS) to demonstrate myelin and its degradation products. Lesions were classified immunohistochemically with respect to demyelinating activity, as previously described [93]. Immunohistochemical analyses used avidin–biotin or alkaline phosphatase/anti-alkaline phosphatase technique without modification [94]. Tissues were exposed (16 hrs, at 4°C), to IgGs specific for: AQP1 (rabbit polyclonal 1:500; Santa Cruz, USA), AQP4 (affinity-purified rabbit polyclonal 1:250; Sigma-Aldrich, USA), myelin proteolipid protein (PLP, rabbit polyclonal 1:500; Serotec, Oxford, USA), 2′3′-cyclic-nucleotide 3′-phosphodiesterase (CNPase, mouse monoclonal 1:2000; Sternberger, USA), myelin oligodendrocyte protein (MOG, mouse monoclonal 1:200; gift of Dr Sarah Piddlesden, Cardiff, UK), myelin-associated glycoprotein (MAG, mouse monoclonal 1:10; Chemicon, USA), glial fibrillary acidic protein (GFAP, mouse monoclonal 1:4000; Dako, Denmark), neurofilament protein (NF, mouse monoclonal 1:800; Dako, Denmark), T lymphocytes (CD3, rat monoclonal 1:400; Serotec, USA), macrophages/microglial cells (KiM1P, mouse monoclonal 1:1000; gift from Dr. Radzun, University of Göttingen, Germany) and activated complement antigen (C9neo, mouse monoclonal 1:200; gift from Dr. Paul Morgan, Department of Biochemistry, Cardiff, UK). Primary antibodies were omitted in control staining. Antigen retrieval was performed as previously described [88].

## Competing interests

Dr. Popescu served as a speaker for Teva Innovation Canada, and receives research support from the Saskatchewan Health Research Foundation (principal investigator) and the Canada Research Chairs program (principal investigator). Dr. Bunyan reports no disclosures. Dr. Guo reports no disclosures. Dr. Parisi serves on scientific advisory boards for the US Government Defense Health Board and the Subcommittee for Laboratory Services and Pathology; serves as a Section Editor for Neurology; receives royalties from the publication of Principles & Practice of Neuropathology, 2nd ed. (Oxford University Press, 2003); and receives research support from the NIH (NS32352-13; co-investigator). Dr. Lennon is a named inventor on a patent relating to AQP4 as a target of pathogenic autoantibodies in NMO and related disorders and on a pending patent related to AQP4 applications to cancer; has received greater than the federal threshold for significant interest from licensing of this technology; receives no royalties from the sale of Mayo Medical Laboratories’ service serological tests; however, Mayo Collaborative Services, Inc., receives revenue for conducting these tests; is named inventor on two patent applications filed by the Mayo Foundation for Medical Education and Research relating to functional assays for detecting NMO/AQP4 antibody; receives research support from the National Institutes of health (NS65829; co-investigator). Dr. Lucchinetti may accrue revenue for a patent re: Aquaporin-4 associated antibodies for diagnosis of neuromyelitis optica; receives royalties from the publication of Blue Books of Neurology: Multiple Sclerosis 3 (Saunders Elsevier, 2010); and receives research support from the NIH (NS49577-R01; principal investigator), the Guthy Jackson Charitable Foundation (principal investigator), and the National MS Society (RG 3185 B-3; principal investigator).

## Authors’ contribution

BFP, YG, JEP and CFL analysed and interpreted the immunohistological data. RFB and CFL collected and interpreted the clinical data. CFL and BFP conceived this study. BFP, CFL and VAL drafted and edited the manuscript. All authors read and approved the final manuscript.
